# Small RNAs Participate in Plant–Virus Interaction and Their Application in Plant Viral Defense

**DOI:** 10.3390/ijms23020696

**Published:** 2022-01-08

**Authors:** Zhiqi Deng, Liqun Ma, Peiyu Zhang, Hongliang Zhu

**Affiliations:** The College of Food Science and Nutritional Engineering, China Agricultural University, Beijing 100083, China; zhiqideng@cau.edu.cn (Z.D.); lqma@cau.edu.cn (L.M.); zhangpy979@163.com (P.Z.)

**Keywords:** small RNA, RNA interfering, antiviral response, viral suppressors of RNA silencing

## Abstract

Small RNAs are significant regulators of gene expression, which play multiple roles in plant development, growth, reproductive and stress response. It is generally believed that the regulation of plants’ endogenous genes by small RNAs has evolved from a cellular defense mechanism for RNA viruses and transposons. Most small RNAs have well-established roles in the defense response, such as viral response. During viral infection, plant endogenous small RNAs can direct virus resistance by regulating the gene expression in the host defense pathway, while the small RNAs derived from viruses are the core of the conserved and effective RNAi resistance mechanism. As a counter strategy, viruses evolve suppressors of the RNAi pathway to disrupt host plant silencing against viruses. Currently, several studies have been published elucidating the mechanisms by which small RNAs regulate viral defense in different crops. This paper reviews the distinct pathways of small RNAs biogenesis and the molecular mechanisms of small RNAs mediating antiviral immunity in plants, as well as summarizes the coping strategies used by viruses to override this immune response. Finally, we discuss the current development state of the new applications in virus defense based on small RNA silencing.

## 1. Introduction

During the long process of co-evolution between plants and viruses, the plant viruses, as obligate parasites with limited genome coding function, need to widely use the host intracellular metabolic system for its replication, expression, and infection establishment, as well as to obtain nutrition for proliferation at the expense of the host [1]. Plant viruses interact deeply with the host in biocycles. The virus first enters the plant cell through the mechanical damage on the plant surface caused by insect vectors or external friction. After entering the cell, the virus particle composed of capsid protein and the virus nucleic acid encapsulated in it disintegrates, then the virus genome will be released and initiate the viral infection period. Viral infection includes the virus replication in the initially infected cells, the transmission of viral particles between cells through plasmodesmata and long-distance transportation through the phloem to other tissues and organs. Finally, the virus will distribute to new hosts. During an infectious attack of plants, the physiological disorders caused by the invasion of viruses are the major limiting factor of agricultural productivity across the world [2]. Viruses account for almost 50% of the pathogens responsible for plant diseases over the world. They can damage natural vegetation and cultivated crops. The economic impact caused by plant viruses exceeds 30 billion dollars annually; the harm of plant virus to crops is not only reflected in yield, but also in quality [3].

In response, plants have evolved a variety of defense pathways to protect themselves, using gene silencing, immune signal transduction, hormone-mediated defense, metabolic regulation and other mechanisms to limit virus replication and movement [1]. Plants activate effector-triggered immunity (ETI) through resistance proteins containing nucleotide-binding leucine-rich repeat domain that recognize viral effectors. This model is similar to the defense mechanism of plants against other non-viral pathogens and it’s one of the main pathogen recognition systems in plants [4]. Another major pathogen recognition system in plants is to use the pattern recognition receptors (PRRs) in plants to recognize the conserved pathogen-associated molecular patterns (PAMPs) and then activate the pattern-triggered immune mechanism (PTI) to limit virus infection [5]. Virus infection may lead to plant hormone disorder, inducing many antagonistic hormones simultaneously and triggering defense response [6]. In addition, the ubiquitin–proteasome pathway is also involved in antiviral defense in plants [7]. In virus–plant interaction, RNA interference (RNAi) is one of the most important mechanisms of plant antiviral immunity. As early as 1928, Wingard et al. discovered that, under some influence, the ringspot visible on the *tobacco* leaves inoculated with *tobacco ringspot virus* (TRSV) would recover. The newly grown leaves were healthy and immune to TRSV when they are infected by TRSV again [8]. This effective mechanism for resisting viruses to restore symptoms and protecting plants from reinfection is RNAi. Small RNAs are the core of RNAi. Small RNAs from different sources can specifically target to inactivate the invading virus and mediate the regulation of endogenous immune genes. Through RNAi-induced virus silencing, plants can specifically degrade virus transcripts at the early stage of virus infection, thus inhibiting the invasion and spreading of the virus [9]. To sum up, plants rely on a variety of innate immunities to recognize the invasion of pathogens and use complex defense mechanisms to resist. This kind of plant–virus combat makes some viruses’ invasion not lead to the occurrence of plant diseases [10].

Small RNAs are a kind of noncoding RNA [11]. Most plant small RNAs are 18–25 nucleotides in length [12]. According to their different origins and biogenesis pathways, small RNAs can be roughly divided into two major categories: microRNAs (miRNAs) and small interfering RNAs (siRNAs). Due to different precursor transcripts and enzymes involved in small RNAs processing, siRNAs have the following subclasses: hairpin-derived small interfering RNAs (hp-siRNAs), secondary siRNAs and heterochromatic siRNAs (hetsiRNAs) [13,14]. Plants have highly diverse small RNAs-based regulatory pathways to meet the various functional requirements of their growth, metabolism and defense [9,13].

## 2. Small RNAs Biogenesis and Classification

### 2.1. Overview of Important Components Involved in Small RNAs Biogenesis

**Figure 1:**
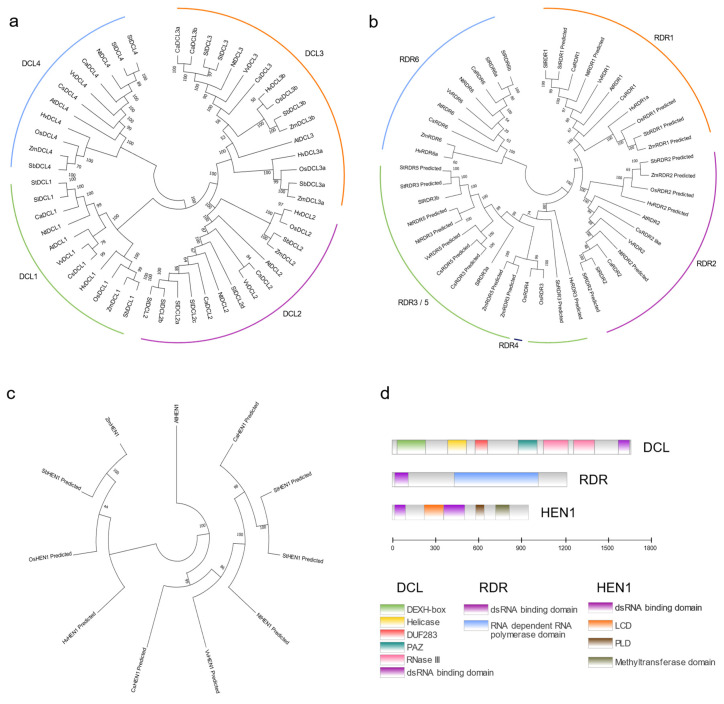
Core components involved in small RNAs biogenesis. (**a**–**c**) Phylogenetic tree based on the alignment of protein sequences of DCLs, RDRs and HEN1 from eleven plant species. (**d**) Schematic representation of the conserved domains in plant DCLs, RDRs and HEN1. The proteins that haven’t been accurately identified are marked with “predicted” after their names. Protein sequences were aligned using MEGA X (https://www.megasoftware.net/) (5 October 2021) to perform the phylogenetic tree. The analysis of the conserved domains was based on TBtools (https://www.yuque.com/cjchen/hirv8i) (5 October 2021). Additional abbreviations: DUF283, domain of unknown function 283; PAZ, PIWI/ARGONAUTE/ZWILLE; LCD, La-motif-containing domain; PLD, PPIase-like Domain. Species names are abbreviated as follows: Sl, Solanum lycopersicum; Nt, Nicotiana tabacum; At, Arabidopsis thaliana; Ca, Capsicum annuum; Sb, Sorghum bicolor; Hv, Hordeum vulgare; Cs, Cucumis sativa; Zm, Zea mays; Os, Oryza sativa; St, Solanum tuberosum; Vv, Vitis vinifera.

Multiple small RNAs produced in plants have different origins and biogenesis pathways. Although small RNAs with different functions involve diverse molecular pathways, several proteins always play important roles in these pathways (Figure 1).

#### 2.1.1. Dicer-like (DCL)

Dicer-like (DCL) proteins are endonucleases of the RNaseIII family, which are important components in small RNAs biogenesis [15]. Members of the RNaseIII family have long been characterized as endonucleases that specifically recognize and process dsRNAs. DCLs dice the dsRNA precursors of small RNAs, then initiate the production of small RNAs [15,16]. The simplest dicer was found in budding yeast, which only contains two domains: RNaseIII domain and dsRNA binding domain(RBD), but lacking the DexD box, helicase domain, domain of unknown function 283 (DUF283) and piwi/Argonaute/ZWILLE (PAZ) domain found in higher eukaryotic DCLs [13,17,18]. Depending on these functional domains, DCLs can recognize and locate dsRNA substrates to achieve the accuracy and specificity of sRNA processing. One mode shows that PAZ and RNaseIII domains play central roles in dsRNA cleavage. The PAZ domain specifically binds to the 2nt-overhang nucleotides at the 3′ end of the dsRNA precursor. The substrate dsRNA extends along the surface of DCL to the cleft in the dimer of two RNaseIII domains. The two strands of the dsRNA will be cleaved by the two RNaseIII active sites, respectively, resulting in staggered duplex ruptures. This structure seems like a ruler determining the size of the small RNA—the length of the small RNA product is dependent on the distance between the PAZ domain and the processing center of DCL [16]. Structural analysis reveals that the DUF283 domain serves as a heterodimer domain for specific dsRNA-binding (DRB) protein interaction [19]. RBD, which exists diversely in different DCLs, participates in the regulation of protein nucleo-cytoplasmic distribution [20,21]. 

Multiple DCL proteins identified in different plant species can be generally classified into four different clades: DCL1 generates 21 and 22nt sRNAs from short stem-loop RNAs, while DCL2, DCL3 and DCL4 produce 22nt, 24nt and 21nt sRNAs from long dsRNA substrates, respectively [22]. DCL1 is mainly involved in the processing of plant miRNAs [23,24]. Plant endogenous siRNAs produced by DCL2, DCL3 and DCL4 are involved in different RNAi pathways. DCL2 and DCL4-dependent secondary siRNAs are the core of post-transcriptional gene silencing (PTGS) regulatory pathway. Secondary siRNAs include several subclasses, such as phased small interfering RNAs (phasiRNAs), trans-acting small interfering RNAs (tasiRNAs), epigenetically-activated small interfering RNAs (easiRNAs) and natural antisense small interfering RNAs (natsiRNAs). Although PTGS involves plenty of small RNAs, heterochromatic siRNAs (hetsiRNAs) produced by DCL3 are the most abundant in plants, which play pivotal roles in the transcriptional gene silencing (TGS) pathway. HetsiRNAs mediate the transcriptional silencing of transposons and pericentromeric repeats through RNA-directed DNA methylation (RdDM) pathways [13,25,26]. In case of virus invasion, DCL4 produces 21nt siRNAs from viral transcripts, thereby playing a major role in primary antiviral defense and DCL2 coordinates with DCL4 by generating 22nt virus-derived siRNAs, which mainly participate in systemic antiviral RNAi [27,28]. However, the latest research found that tomato SlDCL2b can affect the biogenesis of 22nt miRNAs, thus regulating the production of 22nt sec-siRNAs from viral defense genes and finally affecting the virus immunity in tomatoes. This conclusion provides a new idea for the study of the function of DCL2 in plants [29]. DCL3 has been reported to be mainly involved in antiviral defense to DNA viruses; however, its role in RNA virus defense remains unclear [30,31,32].

#### 2.1.2. RNA-Dependent RNA Polymerases (RDRs)

The precursor dsRNAs of siRNAs can be derived from the folding-back of an inverted repeat sequence and the hybridization of transcripts with sequence complementarity and the most commonly, through the function of RDRs [33]. RDRs are mainly involved in the synthesis of plant siRNAs. Under the action of RDRs, single-stranded RNA (ssRNA) and primary siRNA molecules can generate a large number of dsRNAs, which, as the substrates of DCLs, are processed to produce secondary siRNAs, natsiRNAs and hetsiRNAs [11]. RDRs synthesize dsRNAs with or without initial siRNA primers, thus participating in the silencing cascade amplification mediated by primary sRNAs [34,35]. RDR proteins widely exist in natural organisms, they have been found in plants, fungi, RNA viruses and lower animals. RDRs in eukaryotes are mainly divided into three clades: RDRα, RDRβ and RDRγ [36]. The RDRα clade coexists in plants, animals and fungi; the RDRβ clade is unique to animals and fungi; RDRγ has only been found in plants and fungi [36,37]. Plants generally encode multiple RDRs; despite the sequences of RDRs being divergent, they are all defined by the conserved RNA-dependent RNA polymerase catalytic domain, which is the core structural feature of RDRs [17,38].

Taking *Arabidopsis* as an example, *Arabidopsis* encodes six *RDRs*, among which *RDR1*, *RDR2* and *RDR6* belong to the RDRα clade. All of the three contain the C-terminal catalytic DLDGD motif of eukaryotic RDRs [37]. Plants lacking RDR1, RDR2 or RDR6 function will enhance their susceptibility to viruses [39]. However, the function of the RDRα clade in *Arabidopsis* is not singular. Although the three RDRs all take part in the RNAi mechanism, they play major functions in the different pathways and are important participants in RNAi functional diversity. RDR1 mainly regulates the amplification of exogenous siRNAs in plants [37]. RDR6 has a wide range of functions, involving the biogenesis of trans-acting small interfering RNAs (tasiRNAs) and natural-antisense small interfering RNAs (natsiRNAs) in *Arabidopsis* [37]. *Arabidopsis* RDR2 always participates in TGS; in *rdr2* mutant *Arabidopsis* plants, RDR6-dependent transgene PTGS is intensified [40]. This suggests that there may be a competitive mechanism between different RDRs in plants to maintain the balance between different RNA silencing pathways. *Arabidopsis RDR3*, *RDR4* and *RDR5* belong to the RDRγ clade with atypical catalytic DFDGD motif. So far, these RDRs have not been found to have any functions in *Arabidopsis* RNA silencing pathways. The function of the RDRγ clade remains unclear in plants [36,37]. It was found that antiviral defense and viral-siRNA biogenesis still occurred in *rdr1*, *rdr2* and *rdr6* triple mutants in *Arabidopsis* [28], which indicates that they may compose an alternative pathway for antiviral defense.

#### 2.1.3. HUA ENHANCER 1 (HEN1)

The sRNA duplex processed by DCLs needs to be modified by 2′-*O*-methylation at the 3′ terminal to protect the sRNA from being labeled by nucleotidyl transferase HEN1 SUPPRESSOR 1 (HESO1) [41,42], which is a signal that leads to the small RNA degraded by SMALL RNA DEGRADING NUCLEASE 1 (SDN1) [43]. HEN1 was the first sRNA methyltransferase found in *Arabidopsis*. It can methylate miRNA and siRNA duplexes by depositing a methyl onto the 2′-OH of each strand of DCL products to prevent sRNAs from being degraded [44,45]. Plant HEN1 has a highly conserved iconic methyltransferase (MTase) domain different from the commonly known RNA 2′-*O*-MTase. The catalytic site of HEN1 is not the “K-D-K” active site of the RFM superfamily 2′-*O*-MTases; it is also different from the active site of known 2′-*O*-MTases from the SPOUT superfamily [46]. This MTase domain also exists in the homologues of HEN1 in animals [47]. *Arabidopsis* HEN1 contains five domains, four of which can interact directly with the sRNA duplex substrates, including two dsRBD domains, a conserved MTase domain and a La-motif-containing domain (LCD) that can specifically recognize 3′ terminal-OH groups [48]. Another domain that cannot bind to dsRNAs is the PPIase-like domain (PLD), which is similar to the FK506 binding protein found earlier [49].

The co-crystallization analysis of *Arabidopsis* HEN1 and sRNA duplex recombinant complex in vitro showed that HEN1 binds to the dsRNA substrate as a monomer [46]. The two dsRBDs of HEN1 bind to both sides of the A-form small RNA substrate, respectively [50]; the LCD domain binds to the direction of the 3′ end of the unmethylated strand in duplexes as a cap. At the same time, the two-nucleotide overhang at the 3′ end of the methylation strand is wrapped by the active site of the MTase domain [48]. The length selection of HEN1 substrates may be determined by the distance between the MTase region and the capping site of the LCD domain [48]. The loss of 2′-*O*-methylation function in *Arabidopsis* will lead to serious developmental defects, which is speculated to be caused by the excessive degradation of downstream sRNAs [44,51]. At present, the essential role of methylation modification in small RNA biogenesis and function has been clear, but the mechanism of related factors recruitment remains to be studied.

### 2.2. Multiple Ways of Small RNA Biogenesis

#### 2.2.1. The Biogenesis of microRNAs (miRNAs)

The length of most plant miRNAs is 21nt or 22nt. MiRNA biogenesis can be mainly divided into three parts: pri-miRNA transcription and maturation, pre-miRNA processing, mature miRNA processing and modification.

**Figure 2:**
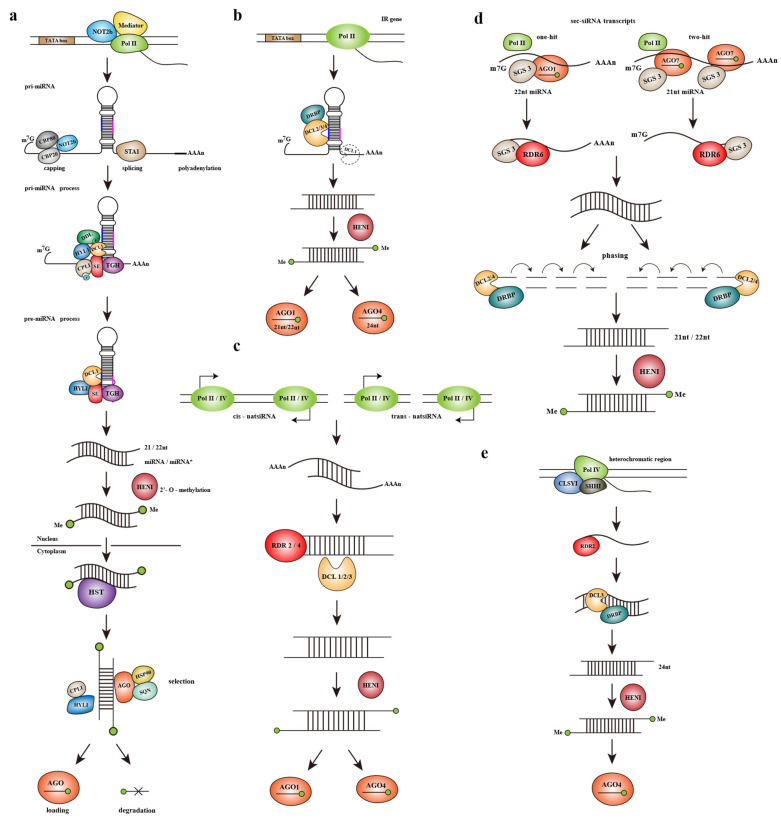
Biogenesis of sRNAs involved in post transcriptional gene silencing and transcriptional gene silencing. (**a**) The micro-RNA (miRNA) pathway. (**b**) The inverted repeat (IR)–derived hairpin-siRNA (hp-siRNAs) pathway. (**c**) The natural-antisense small interfering RNA (natsiRNA) pathway. (**d**) The trans-acting siRNA (tasiRNA) pathway. (**e**) The heterochromatic siRNA (hetsiRNA) biogenesis pathway. Additional abbreviations: m7G, 7-methylguanylate cap; AAAn, polyadenine tail; CBP, CAP-BINDING PROTEIN; STA1, STABILIZED 1; DDL, DAWDLE; DCL, DICER-LIKE; HYL1, HYPONASTIC LEAVES 1; CRL1, C-TERMINAL DOMAIN PHOSPHATASE-LIKE 1; SE, SERRATE; TGH, TOUGH; HEN1, HUA ENHANCER 1; HST, HASTY; AGO, ARGONAUTE; HSP90, HEAT-SHOCK PROTEIN 90; SQN, SQUINT; DRBP, double-stranded RNA-binding protein; SGS3, SUPPRESSOR OF GENE SILENCING 3; RDR, RNA-dependent RNA polymerase; CLSY1, CLASSY 1; SHH1, SAWADEE HOMEODOMAIN HOMOLOG 1; Pol, RNA polymerase; pri-miRNA, primary microRNA; pre-miRNA, precursor microRNA; Sec-siRNA, secondary small interfering RNA; P, phosphate.

Plant miRNA loci are rarely nested in protein coding genes [52]. Pri-miRNA acts as an independent transcription unit to form a fold-back structure, where mature miRNA is generated [52,53]. It can be seen from Figure 2a that pri-miRNA transcripts are transcribed by RNA Polymerase II (Pol II) and require the participation of some coactivators such as Mediator and NOT2b [54]. The production of pri-miRNAs undergoes a series of processing, including capping and polyadenylation [55]. Selectively splicing may occur between the 3′ end and the miRNA fold-back in some of the transcripts, this may improve the accumulation of miRNAs [56,57]. In addition, some related factors involved in pre-miRNA splicing also contribute to pri-miRNA processing. The two subunits CAP-BINDING PROTEIN 80 (CBP80) and CBP20 of cap-binding complex (CBC) and zinc-finger protein SERRATE (SE) all interact with the coactivator NOT2b of pri-miRNA transcription [54,58]. STABILIZED 1 (STA1) also plays an important role in the processing of pri-miRNA, the level of mature miRNA reduced significantly in *sta1*, suggesting that there is a joint defect in pri-miRNA and DCL1 splicing, which is consistent with that shown in *se*, *cbp20* and *cbp80* mutants [59]. *Arabidopsis* plants lacking proline-rich protein SICKLE (SIC) showed a higher level of unspliced pri-miRNA and reduced accumulation of mature miRNA and downstream tasiRNA, which is a typical manifestation of impaired pri-miRNA processing [60].

The pre-miRNA originating from pri-miRNA cleavage is determined by the stem-loop structure in pri-miRNA. The core protein in pri-miRNA cleavage is DCL1, which requires the assistance of HYL1 and SE during its function [58,61]. In vitro experiments showed that HYL1 and SE bind to the dsRNA region and ssRNA/dsRNA junction of pri-miRNA, respectively. Both HYL1 and SE could bind specifically with the DUF283 domain of DCL1. Through the synergy between the three, the accuracy and efficiency of DCL1 cleavage are improved. The knockout mutation of hyl1 will lead to serious developmental defects and loss of function of SE is lethal [61,62]. *Arabidopsis thaliana* loss of RNA-binding protein TOUGH (TGH) shows an increase in the level of pri-miRNA and TGH identified in HYL1 complex is significantly reduced, indicating TGH may also participate in pri-miRNA cleavage by forming a complex with DCL1, HYL1 and SE. It is inferred that the function of TGH may be to regulate the activity of DCL1 [63]. Forkhead-associated-domain protein DAWDLE (DDL) can also bind to pri-miRNA; however, its knockout mutant does not show excessive pri-miRNAs accumulation [64]. DDL may interact with DCL1 through a phosphorylated protein fragment, thereby probably promoting the recognition of pri-miRNA by DCL1. When *DDL* is absent, some pri-miRNAs not integrating with DCL1 will be degraded, leading to a reduced level of pri-miRNAs [65]. Another interactor of SE is C-TERMINAL DOMAIN PHOSPHATASE-LIKE 1 (CPL1). No significant enrichment of unprocessed pri-miRNAs is observed in *cpl*; however, several misprocessed miRNAs appear. There is a model describing the interaction between CPL1 and SE, which guides CPL1 to the DCL1 cleavage complex, thereby CPL1 activates HYL1 through dephosphorylation [66].

The length of plant pre-miRNAs is highly variable. Therefore, different specific processing mechanisms are determined by the sequence processing direction and number of cuts required to acquire mature miRNAs. The processing of the pre-miRNA of stem-loop structure still depends on the DCL1 complex to obtain miRNA/miRNA * duplex after shearing. Upon the release of miRNA/miRNA * and before the duplex dissociation, the 3′ ends of the two strands will be 2′-*O*-methylated by HEN1 (as described above). Since mature miRNAs work in the cytoplasm, the methylated miRNA duplexes will be transported to the cytoplasm by exportin-5 homolog HASTY (HST). The contents of certain miRNAs in nuclear and cytoplasm both decreased in *hst* mutants, indicating that HST may not only mediate miRNAs exportation, but its specific function also requires to be further studied [67].

The selection of the guide strand to be recruited to RISC (RNA-induced silencing complex) to perform its function is determined by its thermodynamic characteristics. Compared with miRNA *, the 5′ end of the guiding strand has lower thermodynamic stability [66,68]. HYL1 and CPL1 also promote this selection. Removing the passenger strand from the miRNA-AGO-RISC complex requires the disassociation among AGO and HSP90 as well as SQN, which may change the conformation of AGO and help successfully release the passenger strand [69,70]. Studies in *M. truncatula* make known that some miRNAs * can also accumulate to a high level in plants and have the ability to cleavage the target transcript and down-regulate its expression [71].

#### 2.2.2. The Biogenesis of Small Interfering RNAs (siRNAs)

- Hairpin-siRNAs (hp-siRNAs)

Hp-siRNAs mainly come from endogenous RNA hairpins. With the help of Pol II, transcripts are obtained from the inverted-repeat (IR) regions scattering in the plant genome. The transcript then forms a hairpin significantly longer than the typical pre-miRNA through extensive base pairing, that is, the precursor of hp-siRNA [72,73]. As shown in Figure 2b, all four DCLs are involved in the biogenesis of hp-siRNAs. Under the cleavage of DCL2/DCL3/DCL4, the precursors can be processed to 22nt/24nt/21nt siRNAs, respectively. DCL1 also takes part in the accumulation of hp-siRNAs indirectly, possibly by promoting the separation of the dsRNA stem from the single-stranded portion of transcripts. The deletion of HYL1, which interacts with DCL1, has little effect on the level of siRNAs. However, the level of DCL3-dependent 24nt siRNA strongly enhanced in the DCL4-interactor drb4 mutant, indicating that there is competition for IR substrates between DCL3 and DCL4-DRB4 complex [72]. The production of siRNAs derived from IRs does not require the RDRs, which always mediate the synthesis of dsRNA precursors in the PTGS pathway. This is consistent with the characteristics of intramolecular base-pairing in IR regions.

The study of *Arabidopsis* hp-siRNAs found that IRs formed in gene replication define a class of rapidly-evolving genes, some of which show stress response, but the biological functions of the large number of endogenous hp-siRNAs are not clear. Because these hp-siRNAs show certain tissue-specific expression and have the ability to drive non-cell-autonomous silencing, it is speculated that they may be involved in stress or environmental signal integration at the whole plant level [72]. The regulatory potential of hp-siRNAs in plants is still ignored so far and entirely annotations of the non-miRNA hairpins are the crux to further in-depth study.

- Natural antisense small interfering RNAs (natsiRNAs)

The precursors of natsiRNAs are dsRNAs formed by annealing of two ssRNA strands transcribed separately but with complementary bases. Complementary transcripts may be produced by Pol II or pol IV. If two complementary ssRNA strands originate from overlapping transcription loci, the natsiRNA products would be called *cis*-natsiRNAs, or they would be called *trans*-natsiRNAs if the precursor ssRNAs come from different loci. *Cis*-NAT pairs exist more widely in plants, accounting for about 9% of the whole *Arabidopsis* genome [74]. However, only 4–6% of *cis*-NAT pairs in Arabidopsis can generate *cis*-natsiRNAs of a high level. It is found that one transcript of the *cis*-NAT pair is usually constitutively expressed and the other may be induced by stress or development. *Cis*-natsiRNAs regulate the expression of constitutive target genes, which endow plants with tolerance to stress [75]. The biogenesis of natsiRNAs requires the participation of a variety of RDRs, which may lead to the cascade amplification effect of siRNA production (Figure 2c). The role of RDRs in natsiRNA biogenesis still needs to be further investigated.

- Secondary small interfering RNAs (sec-siRNAs)

The acquisition of sec-siRNAs is based on miRNA-mediated cleavage. Sec-siRNAs may originate from mRNA transcripts, noncoding-RNAs or transposon sequences, which determines the subclass of sec-siRNAs. At present, the most investigated sec-siRNA biogenesis mechanisms are about the production of tasiRNAs, phasiRNAs and easiRNAs (Figure 2d).

In the corresponding loci of the genome, after transcription by Pol II and a series of modifications such as capping and polyadenylation, the transcript will then be processed by two main pathways to release mature sec-siRNAs [76,77,78]. The “one-hit” pathway mainly involves the participation of 22nt miRNAs or 22nt miRNA variants produced from the monouridylation of 21nt miRNAs. The 22nt miRNA-AGO1 complex first cleaves the Pol II transcript and the miRNA-cleaved 3′ fragment is transformed into dsRNA through RNA-dependent RNA polymerase 6 (RDR6), as well as SUPPRESSOR OF GENE SILENCING 3 (SGS3), then processed by DCL2/DCL4 to obtain 22nt/21nt sec-siRNAs. The two-hit pathway involves 21nt miRNAs and AGO7. The 21nt miRNAs recognize two different targets in the precursor transcript simultaneously and finally cleaved the 3′ target site, so that the final sec-siRNAs come from the 5′ cleavage fragment. The follow-up of the “two-hit” pathway is consistent with the “one-hit”. The single-strand transcripts obtained by the miRNA cleavage would be transformed as the dsRNA substrates of DCL2/DCL4 with the help of RDR6 and the cofactor SGS3, finally obtain the corresponding length sec-siRNAs under the action of DCLs [77,79].

Because of the gradual digestion of DCL2/DCL4, the sec-siRNA productions are often “phased”, that is, their first nucleotide appears every 21 or 22 nucleotides from the miRNA cleavage site [77,79,80]. RDR6 and SGS3 colocalize with AGO in small interfering bodies; it is speculated that SGS3 may also stabilize the interaction between the miRNA-AGO complex and the substrate transcript [81]. The final sec-siRNA products would be loaded to the AGO-RISC complexes to regulate the expression of other protein-coding transcripts.

- Heterochromatic siRNAs (hetsiRNAs)

HetsiRNAs are derived from the heterochromatic regions of the genome. As seen in Figure 2e, Pol IV transcribes the target region of the genome with the help of the SNF2 domain containing protein CLASSY1 (CLSY1) and SAWADEE HOMEDOMAIN HOMOLOG 1 (SHH1) to produce a long ssRNA transcript. After that, the dsRNA substrates processed by RDR2 from the ssRNAs will be cleaved by DCL3 and methylated by HEN1 to produce mature 24nt hetsiRNAs. Mature hetsiRNAs will be loaded to AGO4, then participate in subsequent RNA-dependent DNA methylation (RdDM) pathway [26].

## 3. Virus Defense Based on RNAi

The complete RNAi pathway can be divided into three parts: the biogenesis of sRNAs, target transcripts cleavage of RISC complex guided by the sequence specificity of sRNAs and the transitivity of RNAi. As a natural defense mechanism shared by eukaryotes, RNAi plays a key role in plant resistance to viruses. The core of RNAi antiviral mechanism is a variety of small RNAs produced in RNAi pathway. Small RNAs play different roles in plant defense against viruses according to their different sources and types and a variety of different sRNA antiviral pathways are combined to form a rigorous antiviral network. The following will sum up the antiviral RNAi response in combination with its basic functional pathway.

### 3.1. Virus Derived Small Interfering RNAs Based Splicing of Target Viral Transcripts and Systemic Spread of Antiviral Silencing

**Figure 3:**
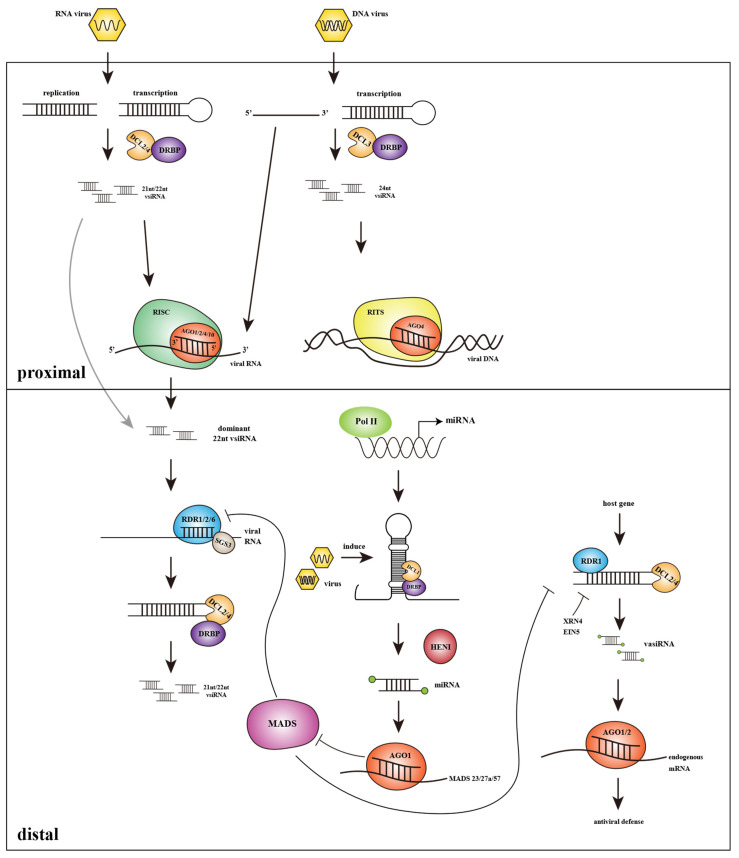
Viral defense pathway driven by host sRNAs and vsiRNAs. Once the virus infects plants, the vsiRNAs generated by DCLs establish the first line of defense in the infected proximal cells by guiding the virus transcript cleavage or mediating the methylation of virus DNA. VsiRNAs will transfer to distal cells and deliver antiviral RNAi with the help of RDRs. In this process, miRNAs and vasiRNAs produced by host plants will participate in the regulation of antiviral defense. Additional abbreviations: DCL, DICER-LIKE; DRBP, double-stranded RNA-binding protein; vsiRNA, virus-derived small interfering RNA; RISC, RNA-induced silencing complex; AGO, ARGONAUTE; RITS, RNA induced transcriptional silencing; Pol II, RNA polymerase II; miRNA, micro RNA; RDR, RNA-dependent RNA polymerase; SGS3, SUPPRESSOR OF GENE SILENCING 3; HEN1, HUA ENHANCER 1; vasiRNA, virus activated small interfering RNA; XRN4, EXORIBONUCLEASE 4; EIN5, ETHYLENE INSENSITIVE 5.

After invading the host cell, the virus first replicates and processes into mature virions by using the host metabolic system. The processed virus particles spread between cells and organs via plasmodesmata and phloem tissue and finally resulting in systemic infection [82,83]. Antiviral RNAi starts from the recognition of viral dsRNA molecules, which are always intermediates of virus replication or arise from secondary RNA-folding [84]. Then the first step after identifying the dsRNA structures is substrates cleavage, which is similar to the synthesis process of plant endogenous siRNAs; host DCLs recognize target viral dsRNAs to produce virus-derived siRNAs (vsiRNAs) of 21–24nt lengths [85]. Next, according to post transcriptional gene silencing (PTGS) pathway, vsiRNAs can be recruited to RNA-induced silencing complex (RISC) associate with AGOs, which mediate posttranscriptional repression of the target viral transcripts through sequence complementary [86,87]. In transcriptional gene silencing (TGS) pathway, vsiRNAs integrate into RNA induced transcriptional silencing (RITS) complex then induce target DNA inactivation through methylation and heterochromatinization [10]. However, the primary vsiRNAs triggered during the replication of RNA viruses or convergent-transcription of DNA viruses don’t play important roles in virus defense, while the transitive secondary vsiRNAs generated via primary vsiRNAs amplification mediated by RDRs are regarded as signals to amplify and spread silencing to effectively respond to systemic infection [35,88]. This systemic silencing generated by secondary vsiRNAs is considered to be a kind of non-cell-autonomous RNA silencing (non-CARS). Different from the cell-autonomous silencing (CARS) in incipient infection cells dominated by DCL4 cleavage, 22nt vsiRNAs produced by DCL2 guide systemic RNA silencing [35,89]. The research on systemic antiviral RNAi shows that the roles of DCL2 and DCL4 are carried hierarchically. DCL2 in distal recipient cells plays an important role in the response of systemic PTGS mobile signals. 22nt vsiRNAs produced by DCL2 will stimulate the host RDRs to amplify the virus dsRNAs, which are the substrates of DCL4 to produce a large number of 21nt secondary vsiRNAs [90]. Through the systemic RNAi pathway, antiviral silencing can be transmitted to the distal region of the plant that is not directly infected. Grafting experiments have shown that 22nt siRNAs are in the center of intercellular RNAi diffusion; they mediate the spread of RNA silencing while other lengths of siRNAs are usually localized within their activities [89,91]. Vazquez et al. Showed that DCL4 plays an inhibitory role in the systemic spread of RNAi; the content of 22nt siRNAs from DCL2 increases in *dcl4* mutant, leading to stronger systemic mobility of PTGS. The amplification of transitivity viral silencing signals in distal tissues also greatly depends on RDRs. RDRs are key factors to synthesize the new dsRNA substrates based on primary siRNAs. In *Arabidopsis* mutants with *RDR6* deletion, there are only primary siRNAs and a lack of secondary siRNAs in plants, which means that silencing can be initiated but is difficult to maintain [88,92]. Under viral existence, the *RDR* deficiency causes enhanced viral toxicity. On the contrary, overexpression of *RDR* in a variety of plants can better protect them from a series of viruses [93]. The TGS pathway against DNA virus is depending on DCL3 and its product 24nt vsiRNAs. 24nt vsiRNAs can induce methylation at the cytosines of viral DNA, thereby weakening the transcription of the virus. RDR2 plays a major role in the amplification of 24nt vsiRNAs in Arabidopsis [8,94]. The above shows that RNAi-mediated viral immunity greatly depends on the presence of amplified vsiRNAs or secondary vsiRNAs (Figure 3).

### 3.2. Role of Endogenous miRNAs Produced in Host Plants in Virus Defense

One of the effects of miRNAs in plant viral defense seems like a bridge between the antiviral RNAi pathway and other host resistance responses (such as ETI/PTI). MiRNAs derived from DCL1 cleavage play a role in regulating endogenous gene expression by loading into the RISC complex to target homologous transcripts in plants [95]. Studies have shown that there are a variety of miRNAs that modulate the immunity genes of ETI and PTI pathways at the posttranscriptional level. *MiR482* binds to the P-loop-NBS-LRR transcripts and results in the silencing of *R* genes. *MiR472* regulates the expression of coiled-coil-NBS-LRR transcripts and in this way to avoid excessive autoimmunity of plants [96,97]. During virus invasion, the expression of the miRNAs related to these viral resistance genes will be downregulated, so as to activate the expression of *NBS-LRR* genes to improve the immune response, such as PTI. The inhibition of miRNAs in the process of plant immunity is often related to the viral suppressors of silencing (VSR, described later) [98].

MiRNAs can also regulate the antiviral RNAi pathway. The targets of *miR444* in rice are MADS-box genes including *MADS57*. MADS57 binds to the CArG motif of RDR1 promoter to inhibit its expression. In the early stage of *Rice stripe virus* (RSV) invasion into rice, it will induce the accumulation of *miR444*, resulting in *MADS57* suppression and thus activate the function of RDR1 [99,100].

### 3.3. Plant Endogenous siRNAs Can Be Induced by Virus and Participate in Antiviral Silencing

Some RDR1-dependent siRNAs will be produced in the plant under viral invasion, these 21nt siRNAs mainly produced by DCL4 are called virus-activated siRNAs (vasiRNAs). VasiRNAs affected by the virus can target multiple endogenous genes and enhance antiviral defense. Their function is to expand the scope of antiviral silencing [101]. The accumulation of vasiRNAs is inhibited by the virus encoding VSR. While CMV lacking VSR-coding gene *2b* infects *Arabidopsis*, the expression of a large number of vasiRNAs is identified in host plants, which decreased significantly in the presence of the 2b suppressor. VasiRNAs are also negatively regulated by endogenous siRNA degradation factors such as EXORIBONUCLEASE 4/ETHYLENE INSENSITIVE 5; this would avoid the overexpression of siRNAs affecting the function of normal genes [101].

### 3.4. Confrontation of the Virus to Host Antiviral RNAi

Plant viruses take measures for their survival. To overcome the host defense mediated by RNAi, viruses encode viral suppressors of RNAi (VSRs) that interfere with the plant antiviral RNAi pathway (Table 1). This can help the virus replicate and spread in the host plant to some extent, so as to gradually realize coevolution in the survival competition with plants. Different viruses encode different VSRs evolved independently. Research showed that they don’t share sequence identity or conserved structure. However, in general, VSRs focus on targeting several critical stages during the antiviral RNAi pathway to interfere with its functions. Several representative common VSRs are briefly introduced below.

The basic strategy of VSRs against RNAi immunity is to disturb the function of RISC to cleavage viral transcripts guided by vsiRNAs. P6 encoded by *Cauliflower mosaic virus* (CaMV) is a translational trans-activator protein, it contains two nuclear localization signals which are crucial to virus infectivity. Haas et al. have shown that one of the functions of P6 is to inhibit the cleavage of DCL4 and finally affect the production of vsiRNAs by interacting with dsRNA binding protein 4 (DRB4), which is an important component of the DCL4 cleavage complex [102]. In addition, P6 also affects the hormone signaling pathway of host plants. By interacting with inactive NPR1 to suppress SA signaling pathway, thereby disrupting SA dependent defense response, making plants more susceptible [103].

**Table 1:** A list of action sites of virus encoded RNA silencing suppressors interfering with host silencing pathway.

Panel Point	Interference Effect	VSRs	Virus	Reference
Small RNA biogenesis	Long dsRNA binding	P14	*Aureus virus*	[104]
		P38	*Turnip crinkle virus*	[105]
		NSs	*Tomato spotted wilt virus*	[106]
		P22	*Tomato chlorosis virus*	[107]
		P21	*Beet yellow virus*	[108]
	DCL blocking	P38	*Turnip crinkle virus*	[105]
		P1	*Rice yellow mottle virus*	[109]
	DRB4 inhibition	P6	*Cauliflower Mosaic Virus*	[102]
	HENI binding	Hc-Pro	*Turnip mosaic virus*	[110]
RISC assemble and function	AGO blocking	P38	*Turnip crinkle virus*	[111]
		2b	*Cucumber mosaic virus*	[112]
		P1	*Sweet potato mild mottle ipomovirus;*	[113]
	AGO degradation	P0	*Polerovirus*	[114]
		P25	*Potato virus X*	[115]
		P38	*Carmo virus*	[111]
		CP	*Nepo virus*	[116]
	sRNA binding	P37	*Pelargonium line pattern virus*	[117]
		P19	*Tomato bushy stunt virus*	[118]
		P21	*Clostero virus*	[104]
		P15	*Peanut clump virus*	[104]
		Hc-Pro	*Poty virus*	[113]
		2b	*Cucumber mosaic virus*	[119]
		NSs	*Tomato spotted wilt virus*	[106]
		NS3	*Rice Stripe Virus*	[120]
		P10	*Grapevine virus*	[121]
		P130	*Tomato mosaic virus*	[105]
		P122	*Tobacco mosaic virus*	[105]
		Rep	*Wheat dwarf virus*	[122]
		P16	*Cucumber vein yellowing virus*	[123]
		PNS10	*Rice Dwarf Phytoreovirus*	[124]
		P14	*Aureus virus*	[104]
Systemic antiviral silencing	RDR6 inactivation	βC1	*Tomato yellow leaf curl China virus*	[125]
		P6	*Rice yellow stunt rhabdovirus*	[126]
		Hc-Pro	*Sugarcane mosaic virus*	[127]
	SGS3 blocking	P2	*Rice stripe virus*	[128]
		V2	*Tomato yellow leaf curl virus*	[116]
		VPg	*Potato virus A*	[129]
		P25	*Plantago asiatica mosaic virus*	[130]
Host miRNA antiviral response	Affect miRNA expression	P19	*Tomato bushy stunt virus*	[131]
		P122	*Tobacco mosaic virus*	[132]
		Hc-Pro	*Poty virus*	[133]
TGS	Interference with TGS	AC2	*Begono virus*	[134]
		Hc-Pro	*Poty virus*	[135]
		AL2	*Tomato golden mosaic virus*	[136]

P19 protein encoded by *tombusvirus* has been intensively studied. Crystal analysis showed that the two P19 monomers are connected at the tail and exist in the form of a homodimer similar to a molecular ruler [118]. Based on its special structure, P19 can selectively bind to the 19 bp length dsRNA region of a siRNA without sequence-specific recognition. In this way, P19 can bind vsiRNAs and inactivate them, thus suppressing vsiRNAs loading onto the RISC complex and preventing viral RNA cleavage mediated by vsiRNAs. On the other hand, P19-vsiRNA binding can also control the diffusion of silencing signal vsiRNAs [118,137]. In addition, P19 restrains plant antiviral defense by affecting the biogenesis of endogenous miRNAs. The activity of P19 can enhance the expression of *miR168*, which regulates the level of its target *AGO1*. In the existence of P19, the viral defense response of host plants will be negatively regulated to help the virus reproduce in plants [131].

The 2b protein of *Cucumber mosaic virus* (CMV) is one of the earliest proteins to be uncovered and studied [138]. Researches show that 2b preferentially colocalized with AGO1 in host cells infected and suppressed the splicing function of AGO1 by interacting with the PAZ domain and PIWI domain of AGO1, so as to interfere with the establishment of antiviral RNAi defense and the subsequent transitivity of systemic RNAi signals. In addition, it was found that the expression of CMV 2b protein in *Arabidopsis* would lead to a phenotype similar to *ago1* mutant, which confirmed that the function of AGO1 is disturbed by 2b under viral invalidation [112]. Crystallographic analysis shows that the 2b protein of *Tomato aspermy virus* (TAV), which is highly homologous with CMV can bind to siRNA duplexes. The analysis of TAV-2b-siRNA complexes indicates that 2b forms a homodimer adopting the α-helical structure, in this way to bind siRNAs by length meaturement [119,139]. This shows that 2b has a dual inhibitory mode, whether inhibiting AGO1 cleavage or hindering siRNAs assembly, the ultimate goal is to prevent the assembling of the RISC complex, so as to prevent the degradation of viral transcripts.

## 4. The Applications of Small RNAs in Plant Viral Defense

The RNAi tactic has been widely used in the study of plant functional genes and disease-related pathogen factors. How to use the RNAi mechanism to improve plant immunity to pathogens has become the major concern of researchers. At present, the main scheme is to improve the resistance of plants to viruses by constructing transgenic plants expressing exogenous artificial sRNAs.

The essence of introducing exogenous sRNAs to improve plant viral defense is to help plants establish a barrier against virus invasions in advance by manufacturing specific artificial sRNA which can target some key genes of host plants and virus transcripts. The targets of these sRNAs can be the susceptible factor transcripts in the host plant. Susceptibility factors are host proteins that play an important role in virus-host interaction. For example, the translation of viral mRNAs depends on the host translation mechanism, so the translation-related protein eIF4E family is a typical susceptible factor. There are several genes encoding different eIF4E subtypes in plants and most viruses use specific eIF4E isoforms for their RNA translation. Therefore, utilizing the functional redundancy between different protein isoforms in plants may realize the precise inhibition of virus-dependent susceptibility factors, but doesn’t affect the normal physiologic function in plants [140]. Another selectable target of artificial sRNA is the key region of viral RNA. The common choices are the RNA regions associated with viral replication and movement along with viral transcripts encoding VSRs. Studies in *Arabidopsis* show that transgenic plants that express exogenous artificial sRNAs targeting multiple VSRs show resistance against multiple viruses [141].

There are a number of methods to construct artificial sRNA vectors and transfer them into plants, the basic principle and mode of action follow the RNAi pathway. There are three methods to induce plants to produce specific sRNAs:

1. Hairpin-loop structure induced silencing. The inverted repeat construct containing the target sequence to be silenced is cloned into the plasmid and transferred into the plant. The dsRNA region is recognized and sliced by plant DCLs to obtain the specific sRNA, which will target and mediate the silencing of host or virus transcript targets under viral invasion [142].

2. Artificial miRNA induced silencing. By constructing a vector carrying pre-miRNA structure in vitro and introducing it into plants, so that pre-miRNA can be expressed in plants and mature miRNAs are generated by plant DCLs to perform subsequent silencing. These pre-miRNAs transported to plants are purposefully designed as complementary sequences of target transcripts and according to the mechanism of miRNA processing, specific artificial miRNA molecules can be obtained in plants [143].

3. TasiRNA induced silencing. The miRNAs directly target the *TAS* locus and guide the cleavage of transcripts to produce phased secondary siRNAs, which are known as tasiRNAs. Based on the generation pathway of tasiRNAs, two methods can be used for the application of tasiRNAs: One is to replace the tasiRNA produced regions in *TAS* loci with siRNAs targeting objective transcripts then transferred to plants. Since the production of tasiRNAs is phased, that is, the *TAS* locus will be sliced off in regular intervals to produce tasiRNAs. Multiple artificial siRNAs can be designed according to the predicted cleavage sites to target several transcripts simultaneously. In the alternate approach, the complete *TAS* loci targeted by a specific miRNA can also be replaced by the long target transcript directly. Thus, after being transferred into the plant, tasiRNAs from the target transcript will be generated through the cleavage of miRNA. Both methods can obtain specific tasiRNAs that target and silence the target transcripts [144]. In addition to the three common artificial sRNA applications above, recent studies have found that miRNAs are not only mobile signals in plants, but also signals among plants, which can realize the communication between distinct plants. The existence of miRNA has been identified in the growth medium of *Arabidopsis* plants; it has been confirmed that the expression of miRNAs in plants is affected by the content of miRNAs in adjacent plants. When miRNAs are added to an exogenous medium, an obvious response is identified in several *Arabidopsis* tissues, especially in roots. Although the mechanism of plant absorption of exogenous miRNA is not clear, these results provide a new way for the application of sRNAs and also show that it is feasible to flexibly use sRNA silencing to construct resistant plants [145].

The possible applications of small RNAs in plant protection is as discussed above. However, although varied methods have been proven feasible in the laboratory, RNAi-based viral defense has not been widely commercialization. We believe that the lack of applications for practical virus prevention and control is mainly due to public perception and regulatory approval system. The resistant plants based on the artificial small RNAs need to be treated by transgenic technology. Due to the lack of safety knowledge involved in this technology, the public’s acceptance of this technology is worrying. The commercialization cost of transgenic crops is high, which is also an important reason for the application being limited. At present, efforts are proceeding for the implementation of RNAi-based plant protection technology and RNAi will play a key role in plant protection in the future.

## 5. Conclusions

Plants are constantly challenged by biotic and abiotic factors in the process of growth, in which biological factors are more invasive and destructive. Virus is one of the main biotic stresses causing a variety of crop diseases. The research on antiviral RNAi evolved in plants is of great significance for the study of plant pathology and the cultivation of resistant crops. Several cases have demonstrated the effect and potential of RNAi in plant pathology. The research on sRNAs involved in RNAi pathway has made considerable progress and continues. Small RNAs provide a strong possibility for mastering the perplexity of cell biology and utilizing its mechanism to improve plant resistance. However, the whole antiviral RNAi pathway is an extremely complex system, which also involves material exchange and signal transmission between plants and viruses. With the continuous emergence of advanced technologies, some new functional sRNAs that participate in antiviral RNAi have been found and more applications of sRNAs are gradually recognized. In order to better study the viral defense response mediated by RNAi, we need to further explore other possible factors involved in the RNAi pathway and reflect on their roles in the viral invasion. In addition, the relationship between the RNAi pathway and other virus resistance pathways deserves more attention, in which sRNAs may play major roles. This part is helpful to understand the complete antiviral system in plants incisively and provides possibilities for the diversified applications of sRNA in virus defense. It should be noted that although some achievements have been made in relevant research, the cultivation of resistant plants based on RNAi is still a long way from a wide range of commercial applications; a better understanding of this field will help RNAi play a key role in plant defense in the future.

## References

[1] Calil I.P., Fontes E.P. (2017). Plant immunity against viruses: Antiviral immune receptors in focus. Ann. Bot..

[2] Khalid A., Zhang Q., Yasir M., Li F. (2017). Small RNA Based Genetic Engineering for Plant Viral Resistance: Application in Crop Protection. Front. Microbiol..

[3] Jones R.A., Naidu R.A. (2019). Global Dimensions of Plant Virus Diseases: Current Status and Future Perspectives. Annu. Rev. Virol..

[4] Hurley B., Subramaniam R., Guttman D.S., Desveaux D. (2014). Proteomics of effector-triggered immunity (ETI) in plants. Virulence.

[5] BBigeard J., Colcombet J., Hirt H. (2015). Signaling Mechanisms in Pattern-Triggered Immunity (PTI). Mol. Plant.

[6] Incarbone M., Dunoyer P. (2013). RNA silencing and its suppression: Novel insights from in planta analyses. Trends Plant Sci..

[7] Alcaide-Loridan C., Jupin I. (2012). Ubiquitin and plant viruses, let’s play together!. Plant Physiol..

[8] Rosa C., Kuo Y.-W., Yan Z., Falk B.W. (2018). RNA Interference Mechanisms and Applications in Plant Pathology. Annu. Rev. Phytopathol..

[9] Prasad A., Sharma N., Muthamilarasan M., Rana S., Prasad M. (2019). Recent advances in small RNA mediated plant-virus interactions. Critical Rev. Biotechnol..

[10] Muthamilarasan M., Prasad M. (2013). Plant innate immunity: An updated insight into defense mechanism. J. Biosci..

[11] Bonnet E., Van de Peer Y., Rouzé P. (2006). The small RNA world of plants. New Phytol..

[12] Ruiz-Ferrer V., Voinnet O. (2009). Roles of Plant Small RNAs in Biotic Stress Responses. Annu. Rev. Plant Biol..

[13] Borges F., Martienssen R.A. (2015). The expanding world of small RNAs in plants. Nat. Rev. Mol. Cell Biol..

[14] Deng P., Muhammad S., Cao M., Wu L. (2018). Biogenesis and regulatory hierarchy of phased small interfering RNAs in plants. Plant Biotechnol. J..

[15] Arikit S., Zhai J., Meyers B. (2013). Biogenesis and function of rice small RNAs from non-coding RNA precursors. Curr. Opin. Plant Biol..

[16] Carthew R.W., Sontheimer E.J. (2009). Origins and Mechanisms of miRNAs and siRNAs. Cell.

[17] Bologna N.G., Voinnet O. (2014). The Diversity, Biogenesis, and Activities of Endogenous Silencing Small RNAs in Arabidopsis. Annu. Rev. Plant Biol..

[18] Wang T., You L., Li R., Fu D.-Q., Zhu B.-Z., Luo Y.-B., Zhu H.-L. (2016). Cloning, identification, and expression analysis of a Dicer-Like gene family from *Solanum lycopersicum*. Biol. Plant..

[19] Qin H., Chen F., Huan X., Machida S., Song J., Yuan Y.A. (2010). Structure of the Arabidopsis thaliana DCL4 DUF283 domain reveals a noncanonical double-stranded RNA-binding fold for protein–protein interaction. RNA.

[20] Muhammad T., Zhang F., Zhang Y., Liang Y. (2019). RNA Interference: A Natural Immune System of Plants to Counteract Biotic Stressors. Cells.

[21] Barraud P., Banerjee S., Mohamed W.I., Jantsch M.F., Allain F.H.-T. (2014). A bimodular nuclear localization signal assembled via an extended double-stranded RNA-binding domain acts as an RNA-sensing signal for transportin 1. Proc. Natl. Acad. Sci. USA.

[22] Hohn T., Vazquez F. (2011). RNA silencing pathways of plants: Silencing and its suppression by plant DNA viruses. Biochim. Biophys. Acta (BBA) Bioenerg..

[23] Mlotshwa S., Schauer S.E., Smith T.H., Mallory A.C., Herr J., Roth B., Merchant D.S., Ray A., Bowman L.H., Vance V.B. (2005). Ectopic DICER-LIKE1 expression in P1/HC-Pro Arabidopsis rescues phenotypic anomalies but not defects in microRNA and silencing pathways. Plant Cell.

[24] Qi Y., Denli A.M., Hannon G.J. (2005). Biochemical Specialization within Arabidopsis RNA Silencing Pathways. Mol. Cell.

[25] Slotkin R.K., Martienssen R. (2007). Transposable elements and the epigenetic regulation of the genome. Nat. Rev. Genet..

[26] Matzke M.A., Mosher R.A. (2014). RNA-directed DNA methylation: An epigenetic pathway of increasing complexity. Nat. Rev. Genet..

[27] Bouché N., Lauressergues D., Gasciolli V., Vaucheret H. (2006). An antagonistic function for Arabidopsis DCL2 in development and a new function for DCL4 in generating viral siRNAs. EMBO J..

[28] Garcia-Ruiz H., Takeda A., Chapman E.J., Sullivan C.M., Fahlgren N., Brempelis K.J., Carrington J.C. (2010). Arabidopsis RNA-dependent RNA polymerases and dicer-like proteins in antiviral defense and small interfering RNA biogenesis during Turnip Mosaic Virus infection. Plant Cell.

[29] Wang T., Deng Z., Zhang X., Wang H., Wang Y., Liu X., Liu S., Xu F., Li T., Fu D. (2018). Tomato DCL2b is required for the biosynthesis of 22-nt small RNAs, the resulting secondary siRNAs, and the host defense against ToMV. Hortic. Res..

[30] Brosseau C., El Oirdi M., Adurogbangba A., Ma X., Moffett P. (2016). Antiviral Defense Involves AGO4 in an Arabidopsis–Potexvirus Interaction. Mol. Plant-Microbe Interact..

[31] Deleris A., Gallego-Bartolome J., Bao J., Kasschau K.D., Carrington J.C., Voinnet O. (2006). Hierarchical Action and Inhibition of Plant Dicer-Like Proteins in Antiviral Defense. Science.

[32] Diaz-Pendon J., Li F., Li W.-X., Ding S.-W. (2007). Suppression of Antiviral Silencing by Cucumber Mosaic Virus 2b Protein in Arabidopsis Is Associated with Drastically Reduced Accumulation of Three Classes of Viral Small Interfering RNAs. Plant Cell.

[33] Axtell M.J. (2013). Classification and Comparison of Small RNAs from Plants. Annu. Rev. Plant Biol..

[34] Baulcombe D.C. (2004). RNA silencing in plants. Nature.

[35] Tang G., Reinhart B.J., Bartel D.P., Zamore P.D. (2003). A biochemical framework for RNA silencing in plants. Genes Dev..

[36] Zong J., Yao X., Yin J., Zhang D., Ma H. (2009). Evolution of the RNA-dependent RNA polymerase (RdRP) genes: Duplications and possible losses before and after the divergence of major eukaryotic groups. Gene.

[37] Willmann M.R., Endres M.W., Cook R.T., Gregory B.D. (2011). The functions of RNA-dependent RNA polymerases in *Arabidopsis*. Arab. Book Am. Soc. Plant Biol..

[38] Venkataraman S., Prasad B.V.L.S., Selvarajan R. (2018). RNA Dependent RNA Polymerases: Insights from Structure, Function and Evolution. Viruses.

[39] Pumplin N., Voinnet O. (2013). RNA silencing suppression by plant pathogens: Defence, counter-defence and counter-counter-defence. Nat. Rev. Genet..

[40] Olmedo-Monfil V., Duran-Figueroa N., Arteaga-Vazquez M.A., Demesa-Arevalo E., Autran D., Grimanelli D., Slotkin R.K., Martienssen R.A., Vielle-Calzada J.-P. (2010). Control of female gamete formation by a small RNA pathway in *Arabidopsis*. Nature.

[41] Zhao Y., Yu Y., Zhai J., Ramachandran V., Dinh T.T., Meyers B.C., Mo B., Chen X. (2012). HESO1, a nucleotidyl transferase in Arabidopsis, uridylates unmethylated miRNAs and siRNAs to trigger their degradation. Curr. Biol..

[42] Ren G., Chen X., Yu B. (2012). Uridylation of miRNAs by HEN1 SUPPRESSOR1 in Arabidopsis. Curr. Biol..

[43] Ramachandran V., Chen X. (2008). Degradation of microRNAs by a Family of Exoribonucleases in *Arabidopsis*. Science.

[44] Yu B., Yang Z., Li J., Minakhina S., Yang M., Padgett R.W., Steward R., Chen X. (2005). Methylation as a Crucial Step in Plant microRNA Biogenesis. Science.

[45] Yang Z., Ebright Y.W., Yu B., Chen X. (2006). HEN1 recognizes 21–24 nt small RNA duplexes and deposits a methyl group onto the 2′ OH of the 3′ terminal nucleotide. Nucleic Acids Res..

[46] Tkaczuk K.L., Obarska A., Bujnicki J.M. (2006). Molecular phylogenetics and comparative modeling of HEN1, a methyltransferase involved in plant microRNA biogenesis. BMC Evol. Biol..

[47] Chen X. (2007). A marked end. Nat. Struct. Mol. Biol..

[48] Huang Y., Ji L., Huang Q., Vassylyev D.G., Chen X., Ma J.-B. (2009). Structural insights into mechanisms of the small RNA methyltransferase HEN1. Nature.

[49] Kang C.B., Hong Y., Dhe-Paganon S., Yoon H.S. (2008). FKBP family proteins: Immunophilins with versatile biological functions. Neurosignals.

[50] Tian B., Bevilacqua P., Diegelman-Parente A., Mathews M.B. (2004). The double-stranded-RNA-binding motif: Interference and much more. Nat. Rev. Mol. Cell Biol..

[51] Chen X., Liu J., Cheng Y., Jia D. (2002). HEN1 functions pleiotropically in Arabidopsis development and acts in C function in the flower. Development.

[52] Griffiths-Jones S., Saini H.K., van Dongen S., Enright A.J. (2007). miRBase: Tools for microRNA genomics. Nucleic Acids Res..

[53] Kim Y.J., Zheng B., Yu Y., Won S.Y., Mo B., Chen X. (2011). The role of Mediator in small and long noncoding RNA production in *Arabidopsis thaliana*. EMBO J..

[54] Wang L., Song X., Gu L., Li X., Cao S., Chu C., Cui X., Chen X., Cao X. (2013). NOT2 Proteins Promote Polymerase II–Dependent Transcription and Interact with Multiple MicroRNA Biogenesis Factors in *Arabidopsis*. Plant Cell.

[55] Xie Z., Allen E., Fahlgren N., Calamar A., Givan S., Carrington J.C. (2005). Expression of *Arabidopsis* MIRNA Genes. Plant Physiol..

[56] Bielewicz D., Kalak M., Kalyna M., Windels D., Barta A., Vazquez F., Szweykowska-Kulinska Z., Jarmolowski A. (2013). Introns of plant pri-miRNAs enhance miRNA biogenesis. EMBO Rep..

[57] Schwab R., Speth C., Laubinger S., Voinnet O. (2013). Enhanced microRNA accumulation through stemloop-adjacent introns. EMBO Rep..

[58] Laubinger S., Sachsenberg T., Zeller G., Busch W., Lohmann J.U., Rätsch G., Weigel D. (2008). Dual roles of the nuclear cap-binding complex and SERRATE in pre-mRNA splicing and microRNA processing in *Arabidopsis thaliana*. Proc. Natl. Acad. Sci. USA.

[59] Ben Chaabane S., Liu R., Chinnusamy V., Kwon Y., Park J.-h., Kim S.Y., Zhu J.-K., Yang S.W., Lee B.-h. (2013). STA1, an Arabidopsis pre-mRNA processing factor 6 homolog, is a new player involved in miRNA biogenesis. Nucleic Acids Res..

[60] Zhan X., Wang B., Li H., Liu R., Kalia R.K., Zhu J.-K., Chinnusamy V. (2012). Arabidopsis proline-rich protein important for development and abiotic stress tolerance is involved in microRNA biogenesis. Proc. Natl. Acad. Sci. USA.

[61] Vazquez F., Gasciolli V., Crété P., Vaucheret H. (2004). The nuclear dsRNA binding protein HYL1 is required for microRNA accumulation and plant development, but not posttranscriptional transgene silencing. Curr. Biol..

[62] Dong Z., Han M.-H., Fedoroff N. (2008). The RNA-binding proteins HYL1 and SE promote accurate in vitro processing of pri-miRNA by DCL1. Proc. Natl. Acad. Sci. USA.

[63] Ren G., Xie M., Dou Y., Zhang S., Zhang C., Yu B. (2012). Regulation of miRNA abundance by RNA binding protein TOUGH in Arabidopsis. Proc. Natl. Acad. Sci. USA.

[64] Yu B., Bi L., Zheng B., Ji L., Chevalier D., Agarwal M., Ramachandran V., Li W., Lagrange T., Walker J.C. (2008). The FHA domain proteins DAWDLE in Arabidopsis and SNIP1 in humans act in small RNA biogenesis. Proc. Natl. Acad. Sci. USA.

[65] Machida S., Yuan Y.A. (2013). Crystal Structure of Arabidopsis thaliana Dawdle Forkhead-Associated Domain Reveals a Conserved Phospho-Threonine Recognition Cleft for Dicer-Like 1 Binding. Mol. Plant.

[66] Manavella P., Hagmann J., Ott F., Laubinger S., Franz M., Macek B., Weigel D. (2012). Fast-Forward Genetics Identifies Plant CPL Phosphatases as Regulators of miRNA Processing Factor HYL1. Cell.

[67] Park M.Y., Wu G., Gonzalez-Sulser A., Vaucheret H., Poethig R.S. (2005). Nuclear processing and export of microRNAs in Arabidopsis. Proc. Natl. Acad. Sci. USA.

[68] Eamens A.L., Smith N.A., Curtin S.J., Wang M.-B., Waterhouse P.M. (2009). The Arabidopsis thaliana double-stranded RNA binding protein DRB1 directs guide strand selection from microRNA duplexes. RNA.

[69] Iki T., Yoshikawa M., Meshi T., Ishikawa M. (2012). Cyclophilin 40 facilitates HSP90-mediated RISC assembly in plants. EMBO J..

[70] Iki T., Yoshikawa M., Nishikiori M., Jaudal M., Matsumoto-Yokoyama E., Mitsuhara I., Meshi T., Ishikawa M. (2010). In Vitro Assembly of Plant RNA-Induced Silencing Complexes Facilitated by Molecular Chaperone HSP90. Mol. Cell.

[71] Devers E., Branscheid A., May P., Krajinski F. (2011). Stars and Symbiosis: MicroRNA- and MicroRNA*-Mediated Transcript Cleavage Involved in Arbuscular Mycorrhizal Symbiosis. Plant Physiol..

[72] Dunoyer P., Himber C., Ruiz-Ferrer V., Alioua A., Voinnet O. (2007). Intra- and intercellular RNA interference in *Arabidopsis thaliana* requires components of the microRNA and heterochromatic silencing pathways. Nat. Genet..

[73] Kasschau K.D., Fahlgren N., Chapman E.J., Sullivan C.M., Cumbie J.S., Givan S.A., Carrington J.C. (2007). Genome-wide profiling and analysis of *Arabidopsis* siRNAs. PLoS Biol..

[74] Wang X.-J., Gaasterland T., Chua N.-H. (2005). Genome-wide prediction and identification of cis-natural antisense transcripts in *Arabidopsis thaliana*. Genome Biol..

[75] Zhang X., Lii Y., Wu Z., Polishko A., Zhang H., Chinnusamy V., Lonardi S., Zhu J.-K., Liu R., Jin H. (2013). Mechanisms of Small RNA Generation from Cis-NATs in Response to Environmental and Developmental Cues. Mol. Plant.

[76] Yoshikawa M., Peragine A., Park M.Y., Poethig R.S. (2005). A pathway for the biogenesis of trans-acting siRNAs in *Arabidopsis*. Genes Dev..

[77] Allen E., Xie Z., Gustafson A.M., Carrington J.C. (2005). microRNA-directed phasing during trans-acting siRNA biogenesis in plants. Cell.

[78] Montgomery T.A., Howell M.D., Cuperus J.T., Li D., Hansen J.E., Alexander A.L., Chapman E.J., Fahlgren N., Allen E., Carrington J.C. (2008). Specificity of ARGONAUTE7-miR390 interaction and dual functionality in TAS3 trans-acting siRNA formation. Cell.

[79] Fei Q., Xia R., Meyers B.C. (2013). Phased, secondary, small interfering RNAs in posttranscriptional regulatory networks. Plant Cell.

[80] Ronemus M., Vaughn M.W., Martienssen R.A. (2006). MicroRNA-targeted and small interfering RNA–mediated mRNA degradation is regulated by Argonaute, Dicer, and RNA-dependent RNA polymerase in *Arabidopsis*. Plant Cell.

[81] Yoshikawa M., Iki T., Tsutsui Y., Miyashita K., Poethig R.S., Habu Y., Ishikawa M. (2013). 3′ fragment of miR173-programmed RISC-cleaved RNA is protected from degradation in a complex with RISC and SGS3. Proc. Natl. Acad. Sci. USA.

[82] Rodriguez-Medina C., Atkins C.A., Mann A.J., Jordan M.E., Smith P.M. (2011). Macromolecular composition of phloem exudate from white lupin (*Lupinus albus* L.). BMC Plant Biol..

[83] Uslu V.V., Wassenegger M. (2020). Critical view on RNA silencing-mediated virus resistance using exogenously applied RNA. Curr. Opin. Virol..

[84] Sharma N., Sahu P.P., Puranik S., Prasad M. (2012). Recent Advances in Plant–Virus Interaction with Emphasis on Small Interfering RNAs (siRNAs). Mol. Biotechnol..

[85] De Ronde D., Butterbach P., Kormelink R. (2014). Dominant resistance against plant viruses. Front. Plant Sci..

[86] Brosseau C., Moffett P. (2015). Functional and Genetic Analysis Identify a Role for Arabidopsis ARGONAUTE5 in Antiviral RNA Silencing. Plant Cell.

[87] Yang Z., Li Y. (2018). Dissection of RNAi-based antiviral immunity in plants. Curr. Opin. Virol..

[88] Vazquez F., Hohn T. (2013). Biogenesis and Biological Activity of Secondary siRNAs in Plants. Scientifica.

[89] Zhang X., Lai T., Zhang P., Zhang X., Yuan C., Jin Z., Li H., Yu Z., Qin C., Tör M. (2018). Mini review: Revisiting mobile RNA silencing in plants. Plant Sci..

[90] Parent J.-S., Bouteiller N., Elmayan T., Vaucheret H. (2014). Respective contributions of *Arabidopsis* DCL2 and DCL4 to RNA silencing. Plant J..

[91] Dalakouras A., Wassenegger M., Dadami E., Ganopoulos I., Pappas M.L., Papadopoulou K. (2020). Genetically modified organism-free RNA interference: Exogenous application of RNA molecules in plants. Plant Physiol..

[92] Melnyk C.W., Molnar A., Bassett A., Baulcombe D. (2011). Mobile 24 nt Small RNAs Direct Transcriptional Gene Silencing in the Root Meristems of *Arabidopsis thaliana*. Curr. Biol..

[93] Wang X.-B., Wu Q., Ito T., Cillo F., Li W.-X., Chen X., Yu J.-L., Ding S.-W. (2010). RNAi-mediated viral immunity requires amplification of virus-derived siRNAs in *Arabidopsis thaliana*. Proc. Natl. Acad. Sci. USA.

[94] Raja P., Wolf J.N., Bisaro D.M. (2010). RNA silencing directed against geminiviruses: Post-transcriptional and epigenetic components. Biochim. Biophys. Acta (BBA) Gene Regul. Mech..

[95] De Felippes F.F., Wang J.-W., Weigel D. (2011). MIGS: miRNA-induced gene silencing. Plant J..

[96] Zhai J., Jeong D.-H., De Paoli E., Park S., Rosen B.D., Li Y., González A.J., Yan Z., Kitto S.L., Grusak M.A. (2011). MicroRNAs as master regulators of the plant NB-LRR defense gene family via the production of phased, trans-acting siRNAs. Genes Dev..

[97] Boccara M., Sarazin A., Thiébeauld O., Jay F., Voinnet O., Navarro L., Colot V. (2014). The Arabidopsis miR472-RDR6 Silencing Pathway Modulates PAMP- and Effector-Triggered Immunity through the Post-transcriptional Control of Disease Resistance Genes. PLoS Pathog..

[98] Li Y., Lu Y.-G., Shi Y., Wu L., Xu Y.-J., Huang F., Guo X.-Y., Zhang Y., Fan J., Zhao J.-Q. (2013). Multiple Rice MicroRNAs Are Involved in Immunity against the Blast Fungus *Magnaporthe oryzae*. Plant Physiol..

[99] Wang H., Jiao X., Kong X., Hamera S., Wu Y., Chen X., Fang R., Yan Y. (2016). A Signaling Cascade from miR444 to RDR1 in Rice Antiviral RNA Silencing Pathway. Plant Physiol..

[100] Lian S., Cho W.K., Kim S.-M., Choi H., Kim K.-H. (2016). Time-Course Small RNA Profiling Reveals Rice miRNAs and Their Target Genes in Response to Rice Stripe Virus Infection. PLoS ONE.

[101] Cao M., Du P., Wang X.-B., Yu Y.-Q., Qiu Y.-H., Li W., Gal-On A., Zhou C., Li Y., Ding S.-W. (2014). Virus infection triggers widespread silencing of host genes by a distinct class of endogenous siRNAs in *Arabidopsis*. Proc. Natl. Acad. Sci. USA.

[102] Laird J., McInally C., Carr C., Doddiah S., Yates G., Chrysanthou E., Khattab A., Love A.J., Geri C., Sadanandom A. (2013). Identification of the domains of cauliflower mosaic virus protein P6 responsible for suppression of RNA silencing and salicylic acid signalling. J. Gen. Virol..

[103] Wang K., Senthil-Kumar M., Ryu C.-M., Kang L., Mysore K.S. (2012). Phytosterols Play a Key Role in Plant Innate Immunity against Bacterial Pathogens by Regulating Nutrient Efflux into the Apoplast. Plant Physiol..

[104] Merai Z., Kerényi Z., Kertész S., Magna M., Lakatos L., Silhavy D. (2006). Double-Stranded RNA Binding May Be a General Plant RNA Viral Strategy To Suppress RNA Silencing. J. Virol..

[105] Burgyán J., Havelda Z. (2011). Viral suppressors of RNA silencing. Trends Plant Sci..

[106] De Ronde D., Pasquier A., Ying S., Butterbach P., Lohuis D., Kormelink R. (2014). Analysis of T omato spotted wilt virus NSs protein indicates the importance of the N-terminal domain for avirulence and RNA silencing suppression. Mol. Plant Pathol..

[107] Landeo-Ríos Y., Navas-Castillo J., Moriones E., Cañizares M.C. (2016). The p22 RNA silencing suppressor of the crinivirus Tomato chlorosis virus is dispensable for local viral replication but important for counteracting an antiviral RDR6-mediated response during systemic infection. Viruses.

[108] Moon J.Y., Park J.M. (2016). Cross-Talk in Viral Defense Signaling in Plants. Front. Microbiol..

[109] Lacombe S., Bangratz M., Vignols F., Brugidou C. (2010). The rice yellow mottle virus P1 protein exhibits dual functions to suppress and activate gene silencing. Plant J..

[110] Sanobar N., Lin P.-C., Pan Z.-J., Fang R.-Y., Tjita V., Chen F.-F., Wang H.-C., Tsai H.-L., Wu S.-H., Shen T.-L. (2021). Investigating the viral suppressor HC-pro inhibiting small rna methylation through functional comparison of HEN1 in angiosperm and bryophyte. Viruses.

[111] Azevedo J., Garcia D., Pontier D., Ohnesorge S., Yu A., Garcia S., Braun L., Bergdoll M., Hakimi M.A., Lagrange T. (2010). Argonaute quenching and global changes in Dicer homeostasis caused by a pathogen-encoded GW repeat protein. Genes Dev..

[112] Zhang X., Yuan Y.-R., Pei Y., Lin S.-S., Tuschl T., Patel D.J., Chua N.-H. (2006). Cucumber mosaic virus-encoded 2b suppressor inhibits Arabidopsis Argonaute1 cleavage activity to counter plant defense. Genes Dev..

[113] Li M.-L., Weng K.-F., Shih S.-R., Brewer G. (2016). The evolving world of small RNAs from RNA viruses. Wiley Interdiscip. Rev. RNA.

[114] Hendelman A., Kravchik M., Stav R., Zik M., Lugassi N., Arazi T. (2013). The developmental outcomes of P0-mediated ARGONAUTE destabilization in tomato. Planta.

[115] Chiu M.H., Chen I.H., Baulcombe D.C., Tsai C.H. (2010). The silencing suppressor P25 of Potato virus X interacts with Argonaute1 and mediates its degradation through the proteasome pathway. Mol. Plant Pathol..

[116] Rahman A., Sinha K.V., Sopory S.K., Sanan-Mishra N. (2021). Influence of virus–host interactions on plant response to abiotic stress. Plant Cell Rep..

[117] Pérez-Cañamás M., Hernández C. (2015). Key Importance of Small RNA Binding for the Activity of a Glycine-Tryptophan (GW) Motif-containing Viral Suppressor of RNA Silencing. J. Biol. Chem..

[118] Vargason J.M., Szittya G., Burgyán J., Hall T.M. (2003). Size Selective Recognition of siRNA by an RNA Silencing Suppressor. Cell.

[119] Chen H., Yang J., Lin C., Yuan Y.A. (2008). Structural basis for RNA-silencing suppression by Tomato aspermy virus protein 2b. EMBO Rep..

[120] Shen M., Xu Y., Jia R., Zhou X., Ye K. (2010). Size-Independent and Noncooperative Recognition of dsRNA by the Rice Stripe Virus RNA Silencing Suppressor NS3. J. Mol. Biol..

[121] Zhou Z.S., Dell’Orco M., Saldarelli P., Turturo C., Minafra A., Martelli G.P. (2006). Identification of an RNA-silencing suppressor in the genome of Grapevine virus A. J. Gen. Virol..

[122] Wang Y., Dang M., Hou H., Mei Y., Qian Y., Zhou X. (2014). Identification of an RNA silencing suppressor encoded by a mastrevirus. J. Gen. Virol..

[123] Valli A., Oliveros J.C., Molnar A., Baulcombe D., García J.A. (2011). The specific binding to 21-nt double-stranded RNAs is crucial for the anti-silencing activity of Cucumber vein yellowing virus P1b and perturbs endogenous small RNA populations. RNA.

[124] Ren B., Guo Y., Gao F., Zhou P., Wu F., Meng Z., Wei C., Li Y. (2010). Multiple Functions of Rice Dwarf Phytoreovirus Pns10 in Suppressing Systemic RNA Silencing. J. Virol..

[125] Li F., Huang C., Li Z., Zhou X. (2014). Suppression of RNA silencing by a plant DNA virus satellite requires a host calmodulin-like protein to repress RDR6 expression. PLoS Pathog..

[126] Guo H., Song X., Xie C., Huo Y., Zhang F., Chen X., Geng Y., Fang R. (2013). Rice yellow stunt rhabdovirus protein 6 suppresses systemic RNA silencing by blocking RDR6-mediated secondary siRNA synthesis. Mol. Plant-Microbe Interact..

[127] Zhang X., Du P., Lu L., Xiao Q., Wang W., Cao X., Ren B., Wei C., Li Y. (2008). Contrasting effects of HC-Pro and 2b viral suppressors from Sugarcane mosaic virus and Tomato aspermy cucumovirus on the accumulation of siRNAs. Virology.

[128] Du Z., Xiao D., Wu J., Jia D., Yuan Z., Liu Y., Hu L., Han Z., Wei T., Lin Q. (2011). p2 of Rice stripe virus (RSV) interacts with OsSGS3 and is a silencing suppressor. Mol. Plant Pathol..

[129] Rajamäki M.-L., Streng J., Valkonen J.P. (2014). Silencing suppressor protein VPg of a potyvirus interacts with the plant silencing-related protein SGS3. Mol. Plant-Microbe Interact..

[130] Aguilar E., Almendral D., Allende L., Pacheco R., Chung B.N., Canto T., Tenllado F. (2014). The P25 Protein of Potato Virus X (PVX) Is the Main Pathogenicity Determinant Responsible for Systemic Necrosis in PVX-Associated Synergisms. J. Virol..

[131] Várallyay É., Válóczi A., Ágyi Á., Burgyán J., Havelda Z. (2010). Plant virus-mediated induction of miR168 is associated with repression of ARGONAUTE1 accumulation. EMBO J..

[132] Csorba T., Bovi A., Dalmay T., Burgyán J. (2007). The p122 subunit of Tobacco Mosaic Virus replicase is a potent silencing suppressor and compromises both small interfering RNA-and microRNA-mediated pathways. J. Virol..

[133] Pruss G.J., Lawrence C.B., Bass T., Li Q., Bowman L.H., Vance V. (2004). The potyviral suppressor of RNA silencing confers enhanced resistance to multiple pathogens. Virology.

[134] Guerrero J., Regedanz E., Lu L., Ruan J., Bisaro D.M., Sunter G. (2020). Manipulation of the plant host by the Geminivirus AC2/C2 protein, a central player in the infection cycle. Front. Plant Sci..

[135] Yang L., Xu Y., Liu Y., Meng D., Jin T., Zhou X. (2016). HC-Pro viral suppressor from tobacco vein banding mosaic virus interferes with DNA methylation and activates the salicylic acid pathway. Virology.

[136] Sanan-Mishra N., Jailani A.A.K., Mandal B., Mukherjee S.K. (2021). Secondary siRNAs in Plants: Biosynthesis, Various Functions, and *Applications* in Virology. Front. Plant Sci..

[137] Ye K., Malinina L., Patel D.J. (2003). Recognition of small interfering RNA by a viral suppressor of RNA silencing. Nature.

[138] Brigneti G., Voinnet O., Li W.X., Ji L.H., Ding S.W., Baulcombe D.C. (1998). Retracted: Viral pathogenicity determinants are suppressors of transgene silencing in *Nicotiana benthamiana*. EMBO J..

[139] Goto K., Kobori T., Kosaka Y., Natsuaki T., Masuta C. (2007). Characterization of Silencing Suppressor 2b of Cucumber Mosaic Virus Based on Examination of its Small RNA-Binding Abilities. Plant Cell Physiol..

[140] Rodríguez-Hernández A.M., Gosalvez B., Sempere R.N., Burgos L., Aranda M.A., Truniger V. (2012). Melon RNA interference (RNAi) lines silenced for Cm-eIF4E show broad virus resistance. Mol. Plant Pathol..

[141] Niu Q.-W., Lin S.-S., Reyes J.L., Chen K.-C., Wu H.-W., Yeh S.-D., Chua N.-H. (2006). Expression of artificial microRNAs in transgenic Arabidopsis thaliana confers virus resistance. Nat. Biotechnol..

[142] Koch A., Biedenkopf D., Furch A., Weber L., Rossbach O., Abdellatef E., Linicus L., Johannsmeier J., Jelonek L., Goesmann A. (2016). An RNAi-Based Control of *Fusarium graminearum* Infections Through Spraying of Long dsRNAs Involves a Plant Passage and Is Controlled by the Fungal Silencing Machinery. PLoS Pathog..

[143] Zhang X., Li H., Zhang J., Zhang C., Gong P., Ziaf K., Xiao F., Ye Z. (2011). Expression of artificial microRNAs in tomato confers efficient and stable virus resistance in a cell-autonomous manner. Transgenic Res..

[144] Jacobs T.B., Lawler N.J., Lafayette P.R., Vodkin L.O., Parrott W.A. (2015). Simple gene silencing using the trans-acting siRNA pathway. Plant Biotechnol. J..

[145] Betti F., Ladera-Carmona M.J., Weits D.A., Ferri G., Iacopino S., Novi G., Svezia B., Kunkowska A.B., Santaniello A., Piaggesi A. (2021). Exogenous miRNAs induce post-transcriptional gene silencing in plants. Nat. Plants.

